# Percutaneous Transcatheter Closure of Multiple Ostium Secundum Atrial Septal Defects: A Case Report

**DOI:** 10.7759/cureus.103732

**Published:** 2026-02-16

**Authors:** Daniel Goshen, Jamil Aboulhosn

**Affiliations:** 1 Ecology and Evolutionary Biology, University of California Los Angeles, Los Angeles, USA; 2 Medicine and Cardiology, University of California Los Angeles David Geffen School of Medicine, Los Angeles, USA

**Keywords:** amplatzer septal occluder, asd (atrial septal defect), cardiology devices, congenital heart disease (chd), transcatheter

## Abstract

Ostium secundum atrial septal defects (ASDs) are a common congenital heart defect that are often asymptomatic until adulthood. Percutaneous transcatheter device closure is now a widely accepted form of treatment as an alternative to surgical repair. A 61-year-old woman presented with two secundum ASDs measuring 17 mm (superior) and 14 mm (inferior) with evidence of significant left-to-right shunting along with right ventricular and right atrial enlargement. Under the guidance of transesophageal echocardiography (TEE), the inferior defect was closed first with an 11 mm Amplatzer septal occluder (ASO) (Abbott Medical Inc., IL, USA), and the superior defect was closed next with an 18 mm ASO. The devices were interdigitated and deployed in a staged fashion to avoid entanglement. Immediate TEE and color Doppler confirmed that both devices were well seated across all rims with no residual shunt. In this case, a two-device strategy using ASOs was required due to the defects being spatially distant from one another. Careful preparation through balloon sizing, TEE guidance, and sequential deployment was taken to minimize the risk of device embolization. This case demonstrates that percutaneous transcatheter device closure through multiple devices is a feasible alternative to a surgical patch in patients with multiple ostium secundum ASDs.

## Introduction

An ostium secundum atrial septal defect (ASD) is one of the more frequent forms of congenital heart defects and is typically located in the fossa ovalis of the atrial septum. They are often a result of incomplete formation or excessive reabsorption of the septum secundum during embryogenesis and can present as a single defect or as multiple separate defects within the septum. During childhood, these defects may be asymptomatic; however, secundum ASD symptoms can emerge with increased age and duration of shunting, where larger or multiple defects can lead to significant left-to-right shunting [1,2]. This may result in serious complications, including right atrial and ventricular volume overload and pulmonary over-circulation, with ultimate outcomes such as arrhythmias, pulmonary hypertension, or even stroke due to paradoxical embolism through the defect(s) [3]. The disorder was previously treated with surgical patch closure, but over the past few decades, it has been commonly treated through percutaneous transcatheter device closure when anatomy is suitable [4]. Current adult congenital heart disease guidelines support this approach in the presence of right-sided chamber enlargement and significant left-to-right shunting (pulmonary-to-systemic blood flow ratio (Qp:Qs) ≥ 1.5:1) [5]. Multiple defects present a technical subset where closure strategy depends on the size of the defect, rim adequacy, and distance between the defects [6]. When they are significantly separated, a two-device approach is considered and may even be required rather than a single occluder strategy. Published and systematic reviews suggest that a multiple-device closure can be practical and effective if guided by careful imaging and defect interrogation [6,7]. This case highlights percutaneous closure of two spatially separated secundum ASDs using a planned two-device strategy with interdigitation while under imaging guidance.

## Case presentation

A 61-year-old woman with two distinct ostium secundum ASDs was referred for cardiac catheterization and percutaneous ASD closure due to progressive dyspnea on exertion, with limitation to climbing one flight of stairs at the time of referral. One defect was found to be smaller and inferior, and the other was larger and superior in the septum. On physical examination, the patient appeared comfortable and in no acute distress. She demonstrated a normal heart and rhythm, with no evidence of murmur; however, she did have fixed splitting of S2. Pulmonary examination revealed clear breath sounds bilaterally. There was no peripheral edema in the extremities. The patient was noted to have significant left-to-right shunting by transthoracic echocardiography (TTE) with evidence of right ventricular and right atrial enlargement. Under general anesthesia, a percutaneous closure procedure was performed on the patient with simultaneous transesophageal echocardiography (TEE) guidance. 

**Figure 1:**
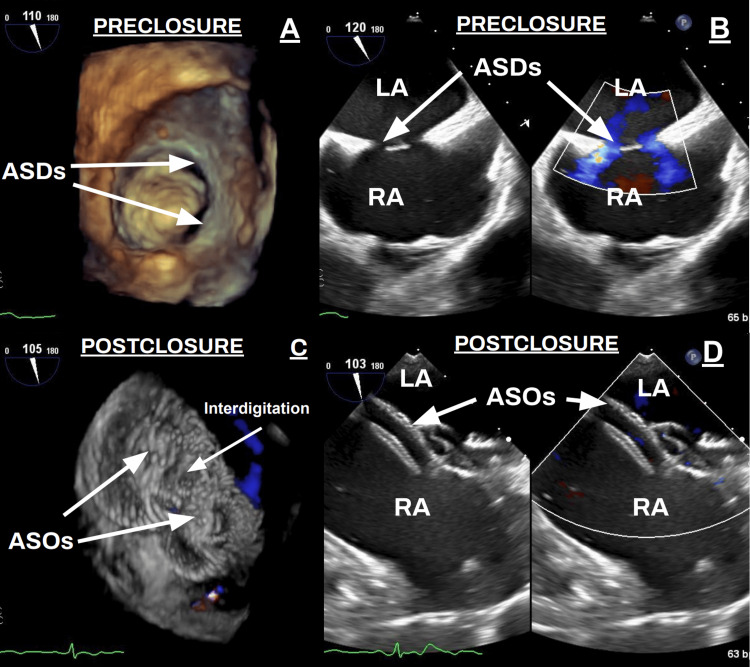
Echocardiograms detailing atrial septal defect (ASD) pre- and post-closure. A: pre-deployment transesophageal echocardiogram showing two ostium secundum defects (arrows); B: pre-closure transthoracic echocardiogram, four-chamber view, and color Doppler depicts shunting through the ASDs (arrows); C: post-closure transesophageal echocardiogram shows two interdigitated ASOs well seated against the septum (arrows); D: post-closure transthoracic four-chamber view with no residual shunt noted (arrows). ASD: atrial septal defect; RA: right atrium; LA: left atrium; ASO: Amplatzer septal occluder

After an 11 Fr sheath was placed in both the right and left femoral veins (RFV, LFV), a right heart catheterization (RHC) was performed with a 7 Fr balloon wedge catheter. Due to the normal wedge pressure, fenestration of the devices was not needed, and the wedge catheter was removed. The TEE demonstrated that the superior and inferior defects measured 17 mm and 14 mm in the largest dimension, respectively (Figure 1A). As the two ASDs were situated distant from each other, it was decided that each defect would be closed separately with its own device (Figure 1A, 1B) [7]. 

After compliant balloon sizing, the smaller, inferior ASD was closed first with an 11 mm Amplatzer septal occluder (ASO) (Abbott Medical Inc., IL, USA) through an 8 Fr Torqueview Trevisio delivery system (Abbott Medical Inc., IL, USA) via the RFV, advanced into the left atrium, and the left atrial disc was deployed. It was then pulled back to the atrial septum, where the right disc was released in the right atrium. The device was kept attached to the cable while the second defect was addressed. The larger, superior ASD was closed next using an 18 mm ASO and was also introduced through an 8 Fr Torqueview Trevisio delivery system. Following the same steps, the device deployed the left atrial disc within the body of the left atrium and was pulled back to the atrial septum to release the right disc in the right atrium. This was done in a manner so that there was interdigitation of both devices (Figure 1C). Push and pull testing was performed to ensure both devices were in a stable position. 

Final TEE and color Doppler confirmed the devices were well seated across all rims, with no residual shunt or pericardial effusion being noted (Figure 1C, 1D). The total fluoroscopy time was 10 minutes without the need for iodinated contrast. The patient remained hemodynamically stable throughout the procedure. She was started on 81 mg of aspirin daily and 75 mg of clopidogrel daily. A congenital TTE was performed the following morning prior to discharge and showed stable device position. The patient returned two weeks post-discharge, and the repeat echo demonstrated device stability and a reduction in right ventricular and right atrial size. 

## Discussion

Multiple ostium secundum ASDs are often monitored conservatively if they only produce minor shunting or are not implicated in embolic events. In adults, the decision to pursue ASD closure is mainly driven by physiologic significance, namely, right atrial/right ventricular enlargement and sufficient net left-to-right shunting [5,8]. In these cases, a closure procedure is needed. This patient experienced significant left-to-right shunting along with right atrial enlargement, supporting transcatheter closure as an appropriate management approach. When multiple ASDs are present, the closure strategy is shaped by anatomy such as morphology, rim adequacy, and the amount of intervening septal tissue [6]. These factors decide whether a single- or two-device strategy is appropriate to complete the closure procedure. Distance between defects, along with balloon interrogation of intervening tissue, has been cited as a significant determinant of which strategy will be used [9]. In this case, a two-device strategy was chosen using ASOs, as the defects were determined to be too distant to close with one device. The staged deployment approach reduced device-device interaction, while occluders were interdigitated to cover both defects while minimizing interference between discs. Published series and systematic reviews support the feasibility of multi-device closure in appropriately selected anatomy [6,7]. Due to the fact that elimination of a long-standing shunt can cause a potential abrupt rise in left atrial pressure, fenestration may be considered [10]. In terms of this procedure, no fenestration of the devices was needed, given that left atrial pressure was not elevated. Balloon sizing and TEE guidance informed device selection and positioning, and stability testing was performed before final release to reduce the risk of malposition or embolization. Imaging confirmed the secured placement of the ASO with no residual shunt. Over time after closure, clinical improvement is often accompanied by right heart remodeling, as previous studies show that a reduction in right-chamber size typically evolves weeks to months after successful closure [11]. At her follow-up two weeks post-intervention, there was already evidence of right heart remodeling with a reduction in the size of both the right atrium and right ventricle, reinforcing the clinical relevance of this approach in appropriately selected anatomy.

## Conclusions

This case demonstrates the feasibility of a planned two-device strategy for percutaneous closure of spatially separated ostium secundum ASDs. The staged deployment with interdigitation under the guidance of TEE resulted in stable placement of the devices with no residual shunt and early reduction of right-sided chamber size on follow-up.
